# Mapping the immune and epigenetic landscape of medication-overuse headache (MOH): a systematic review

**DOI:** 10.3389/fimmu.2026.1756442

**Published:** 2026-01-21

**Authors:** Nanna Elman Andersen, Marta Waliszewska-Prosół, Lanfranco Pellesi

**Affiliations:** 1 Research Unit, Hospital Pharmacy Funen, Odense University Hospital, Odense, Denmark; 2 Department of Neurology, Wroclaw Medical University, Wroclaw, Poland; 3 Clinical Pharmacology, Pharmacy and Environmental Medicine, Department of Public Health, University of Southern Denmark, Odense, Denmark

**Keywords:** epigenetics, immunology, medication-overuse, migraine, opioids, triptans

## Abstract

**Introduction:**

Medication-overuse headache (MOH) develops when drugs intended for acute pain relief lower the threshold for headache chronification. The biological mechanisms driving this transition remain poorly understood. We aim to synthesize current evidence on immune and epigenetic alterations in MOH, identifying new targets of interest and outlining priorities for future research and precision-based interventions.

**Methods:**

We systematically searched PubMed, Embase and Scopus from inception to May 2025 for original human or animal studies reporting immune and/or epigenetic measures in MOH. Risk of bias (RoB) was assessed with the SYRCLE tool for animal studies and JBI tools for human studies. Findings were narratively synthesized, and a domain-based strength-of-evidence (SoE) framework was applied.

**Results:**

Thirteen studies met inclusion criteria. Animal studies identified two immunological pathways involved in MOH resolution: low-dose interleukin-2-mediated expansion of regulatory T cells and inhibition of the P2X purinoceptor 7 (P2X7R)/NLRP3 inflammasome signaling. Clinical studies reported systemic low-grade inflammation in MOH patients, including elevated leukocyte counts, interleukin-6 and gut-derived inflammatory markers. Two epigenetic studies identified differential DNA methylation in genes regulating immune responses and pain transmission. Most studies were small and cross-sectional with limited adjustment. Overall SoE was low-moderate across domains.

**Conclusion:**

Current evidence points to plausible immune and epigenetic involvement in MOH but is insufficient for causal inference or clinical guidance. Findings are hypothesis-generating and most useful for translational prioritization.

## 1 Introduction

Medication-overuse headache (MOH) is a secondary headache disorder that arises from the frequent use of acute headache medications, most commonly triptans, non-steroidal anti-inflammatory drugs (NSAIDs) and opioids (1). MOH is common among individuals with 15 or more headache days per month and up to 90% of patients have a history of either episodic migraine or tension-type headache (2). MOH shows a marked female predominance, with women affected two- to four-fold more often than men. Risk factors for MOH development include high baseline headache frequency, frequent use of acute medications, psychiatric comorbidities such as anxiety and depression, sleep disturbances and socioeconomic vulnerability. Behavioral factors, including maladaptive coping strategies and dependence-like patterns of medication intake, also contribute to disease persistence. Although discontinuation of the overused drug has long been recommended, recent randomized trials and real-world studies show that early initiation of effective preventive therapy without prior withdrawal achieves reductions in headache frequency and can induce remission of medication overuse (3). Nevertheless, many patients continue to experience chronic headache, suggesting that persistent neurobiological alterations may sustain the disorder. Understanding the mechanisms underlying MOH is essential to improve its prevention and management, yet its pathophysiology remains incompletely understood. Recent research has implicated immune dysregulation and epigenetic modifications as potential contributors to MOH development and maintenance (4, 5). Inflammatory mediators and immune cell imbalances may promote central sensitization and hinder resolution of pain. Simultaneously, epigenetic alterations such as deoxyribonucleic acid (DNA) methylation in genes related to immune responses and neuronal plasticity might predispose individuals to MOH or influence their response to treatment. The growing interest in immunological mechanisms has prompted clinical trials of glial modulators in MOH. A randomized, placebo-controlled pilot trial investigated the glial-attenuating agent ibudilast in opioid-related MOH (6). No significant reduction in headache burden was observed, but the study demonstrated that ibudilast reduced peripheral interleukin-1 beta (IL-1β) release following Toll-like receptor stimulation, offering a proof-of-concept that immune modulation may be a viable therapeutic approach. Given the emerging but scattered nature of these findings, we conducted a systematic review to map the current evidence on immune and epigenetic mechanisms involved in MOH. Our aim was to identify consistent patterns across preclinical and clinical studies, highlighting potential therapeutic targets and outlining future research priorities.

## 2 Methods

We performed a systematic literature search to identify original articles reporting the involvement of the immune system and/or epigenetic changes in MOH. The search was conducted in PubMed, Embase (Ovid), and Scopus from database inception to May 2025. We developed and applied search strategies for each database; the full search strings are provided in the **Supplementary Material**. We included both human and animal studies published in English that presented original data. We restricted inclusion to adult populations (≥ 18 years) with a diagnosis of MOH according to International criteria (any version used by the original study) and excluded studies conducted exclusively in children or adolescents. Eligibility was articulated with a PECO orientation for mechanistic evidence: population = adults with MOH; exposure = immunological or epigenetic assessments related to MOH; comparators = healthy controls, episodic or chronic migraine without MOH, or within-subject pre/post contrasts (e.g., before vs. after withdrawal or preventive therapy), when available; outcomes = quantitative immune/epigenetic measures and, secondarily, their associations with headache features or clinical response. We considered cross-sectional, case-control, cohort and pre/post designs. For animal studies, we included vertebrate models explicitly modeling MOH-like states that reported immune or epigenetic outcomes against naïve/sham comparators. Abstracts, reviews, editorials, and other non-original articles were excluded. We also screened the reference lists of relevant studies and articles known to be pertinent by the authors. The review protocol was not registered with PROSPERO or any other public registry. We followed the Preferred Reporting Items for Systematic Reviews and Meta-Analyses (PRISMA) guidelines to ensure transparency and completeness in reporting (7). A completed PRISMA checklist is provided as **Supplementary Material**.

### 2.1 Data extraction

Titles and abstracts of retrieved records were independently screened by two authors for relevance. Full-text articles of potentially eligible studies were reviewed to extract data on study design, populations, methodology and immune parameters assessed. Disagreements were resolved through collegial discussion with the third author. We maintained a log of studies that appeared potentially eligible but were excluded. For each, one primary reason for exclusion was assigned, aligned with our prespecified eligibility criteria. The list of excluded full-text articles with reasons is presented in the **Supplementary Material**. EndNote (version 21.2) was used to manage references and remove duplicates. A flow diagram was created using the PRISMA template, and data were extracted and organized using Excel spreadsheets. The data extraction template is provided in the **Supplementary Material**. We did not contact study investigators because all prespecified data items were available in the published reports and no material ambiguities were identified.

### 2.2 Risk of bias and data synthesis

For animal studies, we assessed internal validity using the SYRCLE Risk of Bias (RoB) tool, which adapts the Cochrane framework for animal experiments (8). For human studies, we assessed RoB with the Joanna Briggs Institute (JBI) critical appraisal tools, selecting the randomized controlled trial checklist for randomized controlled trials, the quasi-experimental checklist for pre-post non-randomized studies and the analytical cross-sectional checklist for cross-sectional studies (9–11). We grouped findings by study type; within clinical studies, we further categorized findings into (i) immunological parameters characterizing patients with MOH; and (ii) changes in epigenetic parameters in patients with MOH. We reserved the option to conduct a meta-analysis if data from sufficiently homogeneous studies became available. Numerical results were reported as in the original publications, typically as mean ± standard deviation (SD).

### 2.3 Certainty/strength of evidence assessment

For each mechanistic outcome, we appraised the strength of evidence (SoE) using an AHRQ-style framework with four levels (high, moderate, low, insufficient). We considered the following domains: study limitations (informed by our risk-of-bias assessments), consistency of direction and magnitude across studies, directness (applicability to MOH-related mechanisms), precision (statistical imprecision/width of confidence intervals or analogous indicators) and potential reporting/publication bias. Because the review integrates human and animal evidence, we assigned separate SoE ratings for human and animal bodies of evidence and then provided an integrated narrative judgment (“Overall SoE”). As a heuristic, human evidence typically starts at “low” and may be upgraded/downgraded based on domains. Animal evidence typically starts at “low” and may be upgraded when multiple models/species and convergent endpoints are present. Two authors independently applied the framework and the third author verified all judgments.

## 3 Results

**Figure 1:**
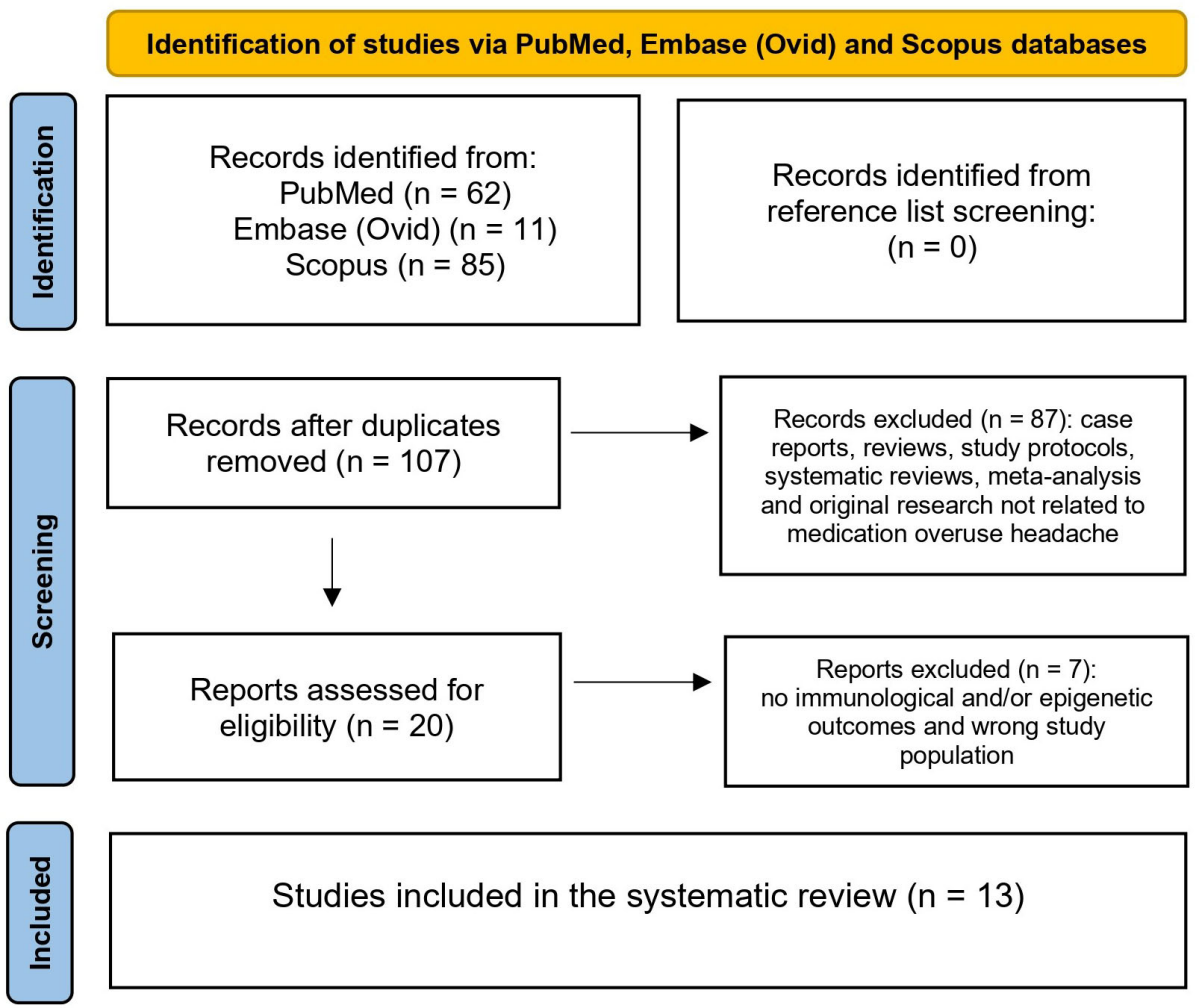
Flowchart of the study.

Our search strategy identified 158 records, of which 13 studies were included in the final review. After excluding 51 duplicate records, 87 were removed because they were case reports, reviews, study protocols, systematic reviews, meta-analyses or original research not related to MOH. Following full-text assessment, 6 additional studies were excluded because they did not report any immunological and/or epigenetic markers and one study was excluded due to age eligibility, as the population comprised only children and adolescents. All discrepancies between the three authors were resolved by consensus. Details for excluded full-text articles are provided in the **Supplementary Material**. In total, 13 studies were included for data synthesis (**Figure 1**).

### 3.1 Animal studies

Animal models of MOH have investigated specific immune alterations associated with this condition. In a mouse model of MOH induced by chronic sumatriptan administration, the resolution of headache-related behavioral sensitization required treatment with low-dose interleukin-2 (IL-2) (12). This effect depended on peripheral chemokine (C-C motif) ligand 2 (CCL2) and chemokine receptor type 2 (CCR2) signaling, which mediated the recruitment of regulatory T cells to the dura mater and trigeminal ganglion. CCL2-CCR2 signaling was not necessary for the development of MOH-like sensitization, but it was essential for its cessation. Another study described that low-dose IL-2 treatment effectively reversed behavioral sensitization in various headache models, including migraine, mild traumatic brain injury and sumatriptan-induced MOH (13). The effect of low-dose IL-2 was independent of mouse sex and strain and did not alter basal nociceptive responses. Regulatory T cells expansion restored immune homeostasis and completely reversed the cutaneous hypersensitivity induced by repeated administration of sumatriptan. In a separate model, repeated sumatriptan exposure led to mechanical hyperalgesia and central sensitization through activation of microglia in the trigeminal nucleus caudalis (14). This process was mediated by the P2X purinoceptor 7 (P2X7R) and the NLR family pyrin domain containing 3 (NLRP3) inflammasome. Pharmacological inhibition of P2X7R or NLRP3 reduced hyperalgesia and the expression of central sensitization markers such as c-Fos and calcitonin gene-related peptide (CGRP). Two additional studies explored the role of peripheral inflammation and gut-brain axis dysfunction (15, 16). In male rats exposed to chronic NSAID treatment (mefenamic acid), elevated serum levels of occludin and lipopolysaccharide-binding protein (LBP) were observed, along with increased brain levels of interleukin-17 (IL-17) and high mobility group box 1 (HMGB1) (15). These changes were associated with pain- and anxiety-like behaviors. In female rats treated chronically with piroxicam, elevated central levels of IL-17, along with increased serum concentrations of CGRP, interleukin-6 (IL-6), and LBP, were reported (16). Methodological aspects and main findings of these animal studies are summarized in **Table 1**.

**Table 1:** Immunological findings in animal models of medication overuse headache (MOH).

Animal	Total number of animals (per group)	Sex	Age	Drug inducing MOH	Immunological findings	Year	Reference
Mouse	6–10 per group	Male and female	8–12 weeks	Daily intraperitoneal injections of sumatriptan succinate (0.6 mg/kg), for 15 days	Treg cells deficit in the trigeminal system during MOH and its reversibility via low-dose IL-2	2020	(13)
	6–9 per group		6–8 weeks	Daily intraperitoneal injections of sumatriptan succinate (0.6 mg/kg), for 11 days	Inhibition of microglia, P2X7R or NLRP3 reduced mechanical hyperalgesia and the expression of c-Fos and CGRP markers in the trigeminal nucleus caudalis	2023	(14)
	6–10 per group		7–10 weeks	Daily intraperitoneal injections of sumatriptan succinate (0.6 mg/kg), for 15 days	Mice with MOH show a reduction of Treg cells in the trigeminal ganglion. Administration of low-dose IL-2 selectively increases Treg cells and resolves the behavioural sensitisation. CCL2-CCR2 signalling is essential for the recruitment of Treg cells in the trigeminal ganglion	2024	(12)
Rat	8 per group	Male	9–10 weeks	Daily intraperitoneal injections of mefenamic acid (20 mg/kg), for 4 weeks	Increased serum levels of occludin and LBP indicated impaired intestinal barrier function, while elevated brain levels of IL-17 and HMGB1 reflected central neuroinflammation. Immunological changes were significantly correlated with mechanical allodynia	2023	(15)
	8 per group	Female	NA	Daily intraperitoneal injections of piroxicam (1 mg/kg), for 30 days	Serum levels of CGRP, IL-6, IL-17, LBP, and occludin were significantly elevated. Corresponding increases in IL-6, IL-17, and HMGB1 were reported in brain tissues, along with strong negative correlations between inflammatory markers and pain thresholds	2023	(16)

CCL2, C-C motif ligand 2; CCR2, C-C chemokine receptor type 2; c-Fos, proto-oncogene c-Fos; CGRP, calcitonin gene-related peptide; HMGB1, high mobility group box 1; IL-2, interleukin 2; IL-6, interleukin-6; IL-17, interleukin 17; LBP, lipopolysaccharide-binding protein; NLRP3, NOD-like receptor protein 3; P2X7R, P2X purinoceptor 7; Treg cell, regulatory T cell.

### 3.2 Immunological aspects of MOH

Clinical studies suggest that MOH is associated with low-grade systemic immune activation. A study examining the G252A polymorphism in the tumor necrosis factor-beta (TNF-β) gene reported that the protective G/G genotype was present in 23% of patients with migraine but completely absent in those with MOH, suggesting that TNF-β-related genetic variation may influence susceptibility to medication-induced disease progression (17). Another study compared 17 migraine patients overusing acute treatments such as triptans or ergotamines with 17 patients with chronic migraine, 17 patients experiencing an acute migraine attack and 17 healthy controls (18). A significantly higher lymphocyte count was observed in MOH patients compared to those with acute migraine (mean ± SD: 2448.7 ± 775.8/mm³ vs. 1859.7 ± 564.7/mm³; *p* = 0.027). The lymphocyte counts in patients with chronic migraine and healthy controls were 2086.1 ± 540.5/mm³ and 1961.7 ± 385.6/mm³, respectively. A subsequent study assessed similar parameters in 29 patients with medication overuse (corresponding to current MOH definitions), 26 patients with episodic migraine without aura and 27 healthy controls (19). White blood cell and lymphocyte counts were significantly higher in patients with chronic migraine and MOH (mean ± SD: 9.386 ± 2.258 and 2.563 ± 796/mm³, respectively) compared to healthy controls (mean ± SD: 6.174 ± 1.429 and 1.790 ± 349/mm³; *p* < 0.01 for both) and patients with episodic migraine (mean ± SD: 5.953 ± 2.715 and 1.680 ± 677/mm³; _p_ < 0.01 for both). CD3^+^, CD4^+^, CD8^+^, and CD19^+^ cell counts were also significantly higher in patients with chronic migraine than in those with episodic migraine and healthy controls. More recently, a study collected blood samples from 120 MOH patients, 29 patients with episodic migraine and 28 healthy controls (20). Approximately 100 MOH patients were followed longitudinally, with blood samples collected at two and six months. The neutrophil-to-lymphocyte ratio (NLR) was significantly higher in MOH patients compared to controls (adjusted *p* < 0.001), indicating a heightened systemic inflammatory response. Furthermore, a reduction in monthly headache days was associated with a decrease in NLR (adjusted *p* = 0.041).

An emerging body of evidence implicates gut–brain axis dysfunction as a contributor to peripheral inflammation in MOH (21, 22). One study evaluated serum samples from 40 patients with NSAID-overuse headache, 35 patients with episodic migraine and 20 healthy controls (21). Serum levels of lipopolysaccharide (LPS), VE-cadherin, hypoxia-inducible factor 1-α (HIF-1α), CGRP and IL-6 were significantly higher in the MOH group compared to both comparison groups. Additionally, levels of LBP and HMGB1 were elevated in MOH patients compared to healthy controls, while IL-17 and occludin levels did not differ significantly between groups. In the second study, serum samples from 32 patients with NSAID-overuse headache and 16 healthy individuals were analyzed (22). Significantly higher levels of LPS, HMGB1, HIF-1α and IL-6 were observed in MOH patients compared to healthy controls. These alterations were associated with an imbalance in the gut microbiota. In a randomized comparative study of triptan-overuse headache, serum IL-6 declined after treatment in the arms receiving greater occipital nerve blocks, whereas withdrawal alone showed no significant change (23). Immunological findings in patients with MOH are summarized in **Table 2**.

**Table 2:** Immunological findings in patients with medication overuse headache (MOH) compared to other study populations.

Patients	Number of patients	Sex	Mean age (SD)	Immunological findings	Year	Reference
MOH	17	Male and female	38.9 (12.8)	A significantly higher lymphocyte count was described in the MOH group compared to patients with migraine without aura (ictal)	2011	(18)
Chronic migraine	17		31.0 (10.4)			
Migraine without aura (ictal)	17		31.4 (9.7)			
Healthy controls	17		36.8 (13.7)			
Chronic migraine and MOH	29		45.7 (11.0)	MOH patients showed an increased white blood cell and lymphocyte count (including CD3^+^, CD4^+^, CD8^+^, and CD19^+^ cells)	2014	(19)
Healthy controls	27		40.9 (8.4)			
Migraine without aura	26		36.8 (9.4)			
Migraine with and without aura	47		36.4 (10.3)	The *G252A* TNF-β gene polymorphism was carried by 23% of the migraine patients but it was absent in MOH patients	2012	(17)
MOH	22		39.6 (9.9)			
MOH	120		42.5 (12.2)	The neutrophil-to-lymphocyte ratio was significantly higher in MOH patients and associated with a reduction in monthly headache days	2023	(20)
Episodic migraine	29		43.1 (10.9)			
Healthy controls	28		42.4 (14.1)			
Triptan-overuse headache	35		36 (9.4)	A decrease in IL-6 levels was observed in repeated GON block administration but not in the group with withdrawal alone	2017	(23)
Triptan-overuse headache	35		38 (10.0)			
Triptan-overuse headache	35		37.0 (9.8)			
Chronic migraine with MOH	32	Female	41.4 (8.5)	Higher serum levels of LPS, HMGB1, HIF-1α and IL-6 were observed in MOH patients	2024	(22)
Healthy controls	16		41.0 (12.1)			
Chronic migraine with MOH	40		38.0 (7.6)	Serum levels of LPS, CGRP, and IL-6 were significantly higher in the MOH group. Levels of LBP and HMGB1 were elevated in MOH patients, while IL-17 levels did not differ between groups	2024	(21)
Episodic migraine	35		35.1 (7.6)			
Healthy controls	20		39.6 (11.1)			

CGRP, calcitonin gene-related peptide; DNA, deoxyribonucleic acid; GON, great occipital nerve; HIF-1α, hypoxia-inducible factor 1-α; HMGB1, high mobility group box 1; IL-6, interleukin 6; IL-17, interleukin 17; LBP, lipopolysaccharide-binding protein; LPS, lipopolysaccharide; SD, standard deviation; TNF-β, tumor necrosis factor-beta.

### 3.3 Epigenetic changes

Epigenetic evidence in MOH is limited but suggests involvement of both immune-related and neuronal plasticity pathways. In an animal model of triptan-induced MOH, histone deacetylase inhibitors panobinostat and givinostat counteracted CGRP overexpression in rats chronically exposed to eletriptan for 1 month (24). Both drugs counteracted hypersensitivity to capsaicin-induced vasodilatation in the trigeminal territory, as well as photophobic behavior and cephalic allodynia. In a pilot genetic study, sequencing of the HDAC3 gene in 23 patients with MOH revealed an association between the rs2530223 polymorphism and the extent of acute medication consumption, suggesting a role in excessive drug use (25). In another study, blood samples were collected from 120 patients with MOH and 57 controls (29 with episodic migraine and 28 healthy individuals) (20). DNA methylation (DNAm) was analyzed in whole blood at baseline and longitudinally in 100 MOH patients after two and six months of treatment. Compared to controls, MOH patients showed higher methylation levels in three genes (CORIN, CCKBR, and CLDN9) in innate immune cells, including neutrophils and natural killer cells. These epigenetic differences did not change significantly over time in relation to headache improvement. In a large longitudinal epigenome-wide association study, blood samples were collected from 98 patients with chronic migraine at baseline and after a 12-week medication withdrawal period (26). DNAm was assessed using whole-blood samples and genome-wide methylation profiling. DNAm differences were significantly associated with response to medication withdrawal treatment in individuals with chronic migraine. A longitudinal change in DNAm at a CpG site within an intron of HDAC4 gene was associated with a reduction in monthly headache days, while baseline DNAm levels at a CpG in the MARK3 gene were associated with a reduction in monthly migraine days at 12 weeks. Methylation levels in HDAC4 significantly decreased over time in responders. In contrast, MARK3 methylation differed between responders and non-responders at baseline and remained stable. Methylation patterns in HDAC4 and MARK3 may serve as epigenetic biomarkers of non-responsiveness or a predisposition to chronification in MOH. Epigenetic findings in patients with MOH are summarized in **Table 3**.

**Table 3:** Epigenetic changes in patients with medication overuse headache (MOH).

Chromosome	Gene	Methylation	Main findings	Year	Reference
5	*HDAC3*	Not evaluated	The G allele of rs2530223 was significantly associated with the number of acute medications/month used and with the number of days/month in which medications were used	2015	(25)
2	*HDAC4*	Reduced over time	Methylation levels in *HDAC4* significantly decreased over time in responders, while *MARK3* methylation differed between responders and non-responders at baseline and remained stable	2023	(26)
14	*MARK3*	Different between responders and non-responders			
4	*CORIN*	Increased	Higher DNA methylation levels in three genes (*CORIN*, *CCKBR* and *CLDN9*) were reported in innate immune cells, including neutrophils and NK cells	2023	(20)
11	*CCKBR*	Increased			
16	*CLDN9*	Increased			

DNA, deoxyribonucleic acid; NK, natural killer.

### 3.4 Risk of bias for animal studies

Reporting was sparse across several SYRCLE domains. No study described methods for random sequence generation, allocation concealment, random housing or random outcome‐assessment order (0/5 unclear each), and baseline characteristics were also unclear in all studies. Blinding of outcome assessors was consistently low risk (5/5), whereas blinding of investigators was low risk in 1/5 and unclear in 4/5. Incomplete outcome data were low risk in 2/5 and unclear in 3/5. Selective reporting was low risk in 1/5 (all prespecified outcomes presented) and unclear in 4/5 due to absence of protocol/registration. “Other bias” was low risk in 1/5 and unclear in the remaining studies. Overall, concerns were driven by unclear risk from underreporting, with isolated strengths in assessor blinding and completeness of outcome data. Detailed item-level ratings are provided in the **Supplementary Material**.

### 3.5 Risk of bias for human studies

Across two quasi-experimental pre-post studies, outcome measurement and statistical analyses were appropriate, but the lack of concurrent controls and non-comparable co-interventions yielded overall moderate risk of bias in both reports. Among cross-sectional/case-control investigations, diagnostic criteria and assay methods were generally well described. However, confounders were seldom identified or controlled (most studies rated “No” or “Unclear” on identification/strategies for confounding), and some analyses were only partially appropriate, resulting in overall moderate to high risk of bias at the study level. In the single randomized trial, randomization was reported, assessor blinding was adequate and outcome measurement was appropriate, but participant/personnel blinding was not feasible, allocation concealment was unclear and attrition/intention-to-treat handling were insufficiently reported, leading to an overall high risk of bias. Full domain-level appraisals are shown in the **Supplementary Material**.

### 3.6 Strength of evidence

Based on the domain-based framework, the SoE for systemic inflammation in MOH is low-moderate in humans, supported by multiple studies showing elevations in leukocyte-based indices and IL-6 (18–20, 22, 23). Animal evidence for this specific mechanism was insufficient, yielding an overall low rating. Epigenetic signals are supported by low SoE in humans by small, heterogeneous cohorts with limited animal evidence, resulting in overall low SoE (20, 24–26). For low-dose IL-2 mediated T-reg expansion and reversal of MOH-like sensitization, human evidence is not available, while animal evidence is moderate and replicated across models with coherent biological plausibility (12, 13). The overall rating is low due to the translational gap. For central neuroinflammation via P2X7R/NLRP3, no human studies were identified. A single animal study supports low SoE (14), with overall low certainty and uncertain clinical correlates. **Table 4** describes mechanism-specific ratings and limitations.

**Table 4:** Strength of evidence (SoE) for immune and epigenetic mechanisms in medication-overuse headache (MOH).

Mechanism	Human evidence	Human SoE	Animal evidence	Animal SoE	Overall SoE	Limitations
Systemic inflammation (high IL-6 levels, high number of leukocytes and increased neutrophil-to-lymphocyte ratio)	Multiple studies report elevations with consistent direction of effect	Low-moderate	Insufficient	NA	Low	Small samples, mostly cross-sectional. There was limited adjustment for comorbidities/medications
Epigenetic signals (the rs2530223 polymorphism, reduced *HDAC4* methylation over time, baseline *MARK3* difference between responders and non-responders and DNAm in *CORIN*/*CCKBR*/*CLDN9*)	Heterogeneous, early-phase findings across small cohorts	Low	Limited	NA	Low	There are multiple-comparison concerns and replication is lacking
Low-dose IL-2 expands Treg and resolves MOH-like sensitization	Not assessed in humans	NA	Replicated across models with coherent biological plausibility	Moderate	Low	Translational gap (no human studies to date)
Central neuroinflammation via P2X7R/NLRP3	Not assessed in humans	NA	A single study with convergent findings	Low	Low	A single animal study with uncertain clinical correlates

IL-2, interleukin 2; IL-6, interleukin 6; NA, not applicable; NLRP3, NOD-like receptor protein 3; P2X7R, P2X purinoceptor 7; Treg, regulatory T cell.

## 4 Discussion

**Figure 2:**
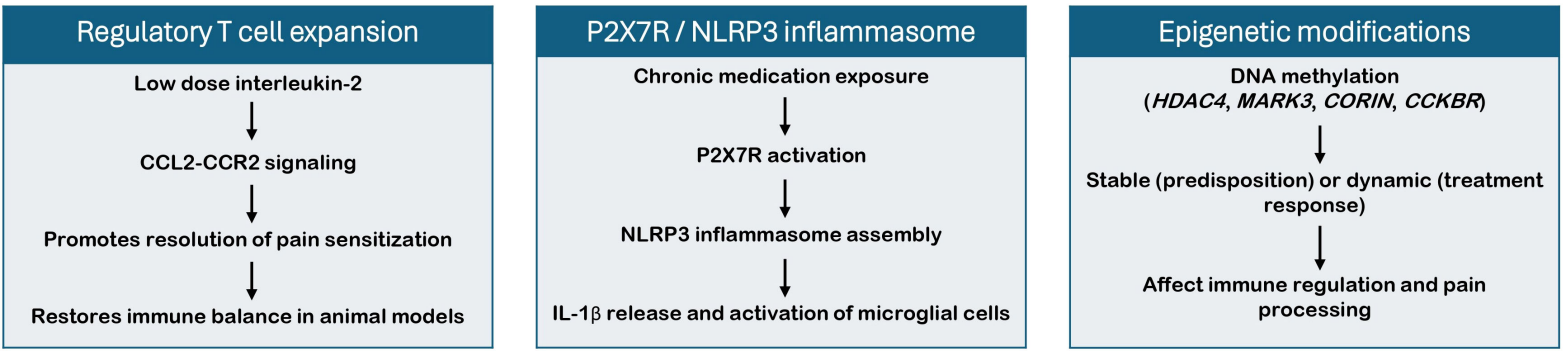
Immune and epigenetic pathways implicated in medication-overuse headache (MOH).

MOH is increasingly viewed within an immunological framework, which reflects its complex interplay with migraine (**Figure 2**). MOH adds to the burden of migraine and significantly increases both individual disability and healthcare costs (1). This complexity calls for a therapeutic paradigm that prioritizes timely preventive treatment (including CGRP-pathway agents and other evidence-based options), with medication withdrawal individualized rather than prescriptive, while in parallel advancing the identification of novel biomarkers and drug targets. In the following sections, we outline pharmacological considerations derived from the immune and epigenetic findings summarized in this review, which should be interpreted as translational and hypothesis-generating. A consistent finding across studies included in this review is the presence of low-grade systemic inflammation: patients with MOH show higher total leukocyte and lymphocyte counts compared to patients with episodic migraine and healthy controls, along with elevated serum levels of IL-6 (18, 19, 21). IL-6 sensitized peripheral nociceptors and facilitated central transmission, and its levels remained high during migraine interictal periods (27–29). Pharmacologically, three classes of IL-6 blockade are available: two anti-IL-6 receptor antibodies (tocilizumab, sarilumab) and an anti-IL-6 antibody (siltuximab). However, none has been tested in chronic pain states. IL-17 is another cytokine implicated in neuro-immune crosstalk that is upregulated in pre-clinical models of migraine and other chronic pain conditions (30–32). Animal models of MOH described the upregulation of IL-17, although evidence in patients remains inconclusive (15, 16, 21). A portfolio of IL-17-targeting biologics is licensed for psoriasis and spondyloarthropathies (secukinumab, ixekizumab, bimekizumab and brodalumab). Their safety and immunomodulatory efficacy make them attractive candidates for repurposing, although clinical data in pain conditions are scarce (33). Beyond circulating cytokines, accumulating evidence points to a dysfunctional gut-brain axis. Increased serum LPS, LBP, HMGB1 and HIF-1α indicate an impaired intestinal-barrier integrity (“leaky gut”). Such microbiota-driven peripheral inflammation can amplify trigeminal excitability and promote headache chronification. In MOH, the immuno-inflammatory environment in the gut may be a pathogenic driver rather than a secondary phenomenon. Growing insight into microbiome modulation in Alzheimer’s and Parkinson’s diseases underscores the translational relevance of targeting gut-brain immune pathways, providing conceptual support for exploring similar mechanisms in MOH (34, 35).

### 4.1 Animal findings: new targets?

Animal studies offer a finer‐grained perspective on causality and have uncovered two immunological pathways or cascades of particular interest. First, low-dose IL-2 expanded regulatory T cells and downregulated the CCL2–CCR2 chemokine axis, thereby reversing sumatriptan-induced cutaneous sensitization in rodents (12, 13). Although IL-2 exhibits antinociceptive activity in both central and peripheral sites, its plasma half-life is measured in minutes and sustained delivery will therefore require engineered IL-2 muteins, pegylation or targeted carriers (36, 37). The second pathway centers on the P2X7R, which is activated by extracellular adenosine triphosphate (ATP) released during cellular stress (38). In mouse models of migraine, either genetic deletion or pharmacological inhibition of P2X7R prevented nitroglycerin-evoked mechanical and thermal hyperalgesia (39–41). Of interest, P2X7R activation promoted assembly of the NLRP3 inflammasome, an intracellular signaling platform that drives IL-1β release and microglial activation (42). NLRP3 is upregulated in microglial cells of the trigeminal nucleus caudalis after exposure to nitroglycerin, which implicates this complex in pain sensitization (43). To date, no P2X7R or NLRP3 inhibitors are licensed in Europe. Several small-molecule antagonists (e.g., AZD9056 and JNJ-54175446 for P2X7R; dapansutrile and tranilast for NLRP3) have shown favorable safety profiles and robust anti-inflammatory effects in early clinical trials for immune and neurodegenerative disorders. Their ability to modulate innate immune signaling makes them plausible candidates for repurposing in chronic pain states once efficacy and long-term safety in other populations are established.

### 4.2 Epigenetic targets

Epigenomic studies in MOH are only a few, yet they show that DNAm changes touch both immune-related loci, such as CORIN, CCKBR and CLDN9, and neuronal-plasticity genes, including HDAC4 and MARK3. Altered methylation at CORIN, CLDN9 and MARK3, genes known for blood-pressure control, gut-barrier integrity and cytoskeletal dynamics, may signal systemic disturbances that favor headache chronification rather than pain transmission per se (44–46). By contrast, methylation at CCKBR, which encodes the cholecystokinin-B receptor, could influence pain directly because antagonism of this receptor reduces hyperalgesia in animal models (47). Likewise, the subcellular trafficking of HDAC4 in dorsal-horn neurons has been linked to lower pain thresholds; when HDAC4 leaves the nucleus, chromatin around pro-nociceptive genes opens and sensitivity increases (48). These observations suggest the existence of two layers of epigenetic regulation in MOH. Some methylation marks appear stable and may identify individuals predisposed to chronification, whereas others fluctuate with disease activity and could serve as treatment biomarkers. This pattern has practical relevance because several classes of “epidrugs” already modulate the same pathways in other diseases (49). Broad histone-deacetylase (HDAC) inhibitors such as valproate and vorinostat, DNA-methyltransferase (DNMT) inhibitors such as azacitidine and decitabine, and BET-bromodomain blockers like apabetalone all dampen transcription of inflammatory or pronociceptive genes (50–52). More selective compounds that degrade or inhibit the HDAC4 protein are in pre-clinical development (53). In MOH, peripheral methylation profiles should be explored as biomarkers to stratify patients by risk and to monitor response to withdrawal therapy. Mechanism-based trials of HDAC, DNMT or BET inhibitors (alone or as adjuncts) are warranted to test whether targeted epigenetic modulation can alleviate MOH.

### 4.3 Causality and treatment implications

Multiple clinical studies report leukocyte-based indices and circulating cytokines consistent with low-grade systemic inflammation in MOH, but the human evidence is predominantly cross-sectional and therefore does not establish temporal directionality. Immune alterations could represent: (1) a predisposing phenotype that increases susceptibility to headache chronification under repeated acute medication exposure; (2) a consequence of medication overuse itself; or (3) an epiphenomenon of chronic pain. Limited longitudinal evidence suggests that inflammatory indices may track clinical improvement (e.g., reductions in headache frequency paralleling reductions in inflammatory markers), which supports biological state-dependence but does not prove causality. In contrast, preclinical models provide more direct support that immune pathways can modulate MOH-like sensitization, but translational inference remains constrained by differences in exposure paradigms and endpoints. Direct evidence that immune modulation improves MOH is limited. A proof-of-concept randomized placebo-controlled pilot trial of the glial attenuator ibudilast in opioid-related MOH did not demonstrate a significant reduction in headache burden, but it did show modulation of immune responsiveness (reduced IL-1β release following Toll-like receptor stimulation), supporting the feasibility of targeting neuroimmune pathways in MOH (6). In addition, in triptan-overuse headache, greater occipital nerve block has been associated with reductions in circulating IL-6 in treatment arms using repeated blocks, suggesting that some interventions may influence inflammatory tone alongside clinical outcomes (23). Beyond these limited interventional data, animal models indicate that expanding regulatory T cells with low-dose IL-2 can reverse MOH-like sensitization, and that inhibiting P2X7R/NLRP3 inflammasome signaling can attenuate central sensitization (12–14). These findings motivate translational prioritization of immune-targeting strategies, while recognizing that clinical efficacy and safety in MOH remain unproven.

### 4.4 Limitations and future perspectives

This review has several limitations. First, peripheral inflammatory and epigenetic markers offer only an indirect view of neuro-inflammatory processes within the trigeminal system. The assumption that systemic signals mirror trigeminal pathology is not established by the included studies. To mitigate this, we separated human and animal evidence, framed conclusions as hypothesis-generating, and avoided causal language. Second, most human data are observational (often cross-sectional), limiting temporality and precluding causal inference. Whether these changes precede medication overuse, arise as a consequence of repeated drug exposure, or represent epiphenomena of chronic headache remains unresolved. Third, assay and outcome heterogeneity precluded meta-analysis. We harmonized units where possible and relied on narrative synthesis, which can dilute consistency and precision. Fourth, several mechanistic domains have small evidence bases, precluding formal small-study assessments and raising concern for publication/reporting bias. Fifth, the translational gap from animal models to humans remains uncertain. We conducted formal risk-of-bias assessments and used these to downgrade certainty where appropriate. Taken together, the overall quality/certainty of evidence is low to moderate, supporting mechanistic hypotheses and translational prioritization rather than clinical recommendations. Finally, our synthesis suggests broadly similar immune and epigenetic alterations across studies, but the parameters analyzed may differ depending on the overused substance. The limited number of available studies and their heterogeneity preclude firm conclusions. Future investigations should compare immune and epigenetic profiles according to the class of medication overused, as substance-specific mechanisms might explain differential susceptibility to chronification and response to withdrawal.

### 4.5 Sex and gender considerations

The current evidence has limitations regarding sex and gender. Clinical cohorts are not powered for sex-stratified analyses, sex distributions are frequently imbalanced (reflecting the higher prevalence of MOH among women), and most studies do not report outcomes stratified by sex or evaluate gender-related determinants. In the preclinical literature, models vary in whether both sexes are included, and where both sexes are studied, sex-specific effects are seldom examined systematically. As a result, it remains unclear whether the reported immune and epigenetic signals differ by sex, whether hormonal status modifies inflammatory trajectories in MOH, or whether sex/gender-related factors confound observed associations. Future studies should prespecify sex-stratified analyses, report hormonal/menopausal status where feasible, and consider gender-related behavioral contributors to medication overuse and chronification.

## 5 Conclusion

Current evidence supports plausible immune and epigenetic involvement in MOH, with signals of low-grade systemic inflammation and preliminary methylation changes in humans. Convergent data on T-reg expansion and microglial inflammasome activity have been described in animal models of MOH. The overall certainty is low to moderate, limited by small, largely cross-sectional human samples. These findings are hypothesis-generating and should inform translational prioritization, not clinical recommendations. Future work should: 1) be adequately powered, longitudinal and preregistered; 2) use harmonized assays and central nervous system readouts where feasible; 3) address confounding; 4) test mechanism-based interventions to validate biomarkers and clarify causality.

## Data Availability

The original contributions presented in the study are included in the article/**Supplementary Material**. Further inquiries can be directed to the corresponding author/s.
