# Supplementary material for: Mapping the immune and epigenetic landscape of medication-overuse headache (MOH): a systematic review
Source: Front Immunol. 2026 Jan 21;17:1756442. doi: 10.3389/fimmu.2026.1756442 (PMC12867861; doi:10.3389/fimmu.2026.1756442)
Supplement: Supplementary file 1 [file DataSheet1.docx]

Supplementary Material

# Supplementary Figures and Tables

## Supplementary Tables

**Supplementary Table 1**. Search strategies utilized for the systematic search.

| **Database** | **Search strategy** |
| --- | --- |
| PubMed | ("medication overuse headache"[tiab] OR "medication-overuse headache"[tiab] OR ("medication overuse"[tiab] AND headache[tiab]) OR "rebound headache"[tiab] OR MOH[tiab]) AND ("neuroinflammation"[tiab] OR "immune response"[tiab] OR "cytokines"[tiab] OR "inflammatory markers"[tiab] OR epigenetic*[tiab] OR "DNA methylation"[tiab] OR methylat*[tiab] OR "histone*"[tiab] OR chromatin[tiab] OR microRNA*[tiab] OR miRNA*[tiab] OR lncRNA*[tiab]) |
| Embase (Ovid) | ((medication OR drug OR analgesic OR triptan OR ergotamine).ti,ab  AND (overuse OR overused OR overusing OR misuse OR dependence OR addiction).ti,ab AND (headache OR migraine).ti,ab) AND ((cytokine OR interleukin OR tumor necrosis factor OR c-reactive protein OR neuroinflammation OR immune dysregulation OR inflammation).ti,ab OR (epigenetic* OR epigenom* OR "DNA methylation" OR methylat* OR "histone acetyl*" OR "histone deacetyl*" OR HDAC* OR DNMT* OR chromatin OR "chromatin remodel*" OR microRNA* OR miRNA* OR "noncoding RNA*" OR "long noncoding RNA*" OR lncRNA*).ti,ab) |
| Scopus | TITLE-ABS-KEY("medication overuse headache" OR "medication-overuse headache" OR ("medication overuse" W/2 headache) OR (MOH W/2 headache)) AND TITLE-ABS-KEY(neuroinflamm* OR "immune response" OR cytokine* OR interleukin* OR epigenetic* OR "DNA methylation" OR methylat* OR "histone*" OR chromatin OR microRNA* OR miRNA* OR lncRNA*) |

**Supplementary Table 2**. Template for data extraction.

| First author | Year of publication | Journal | Study design | Healthy volunteers | Individuals with episodic migraine | Individuals with chronic migraine and/or MOH | Headache or headache-free periods | Immunological parameters | Main findings |
| --- | --- | --- | --- | --- | --- | --- | --- | --- | --- |
|  |  |  |  |  |  |  |  |  |  |

**Supplementary Table 3**. Full-text articles excluded and primary reasons for exclusion.

| **Study** | **Study design** | **Study population** | **Primary reason for exclusion** |
| --- | --- | --- | --- |
| Fumal et al., Brain 2006 | Prospective observational study | Patients with chronic migraine and medication-overuse headache | No immunological/epigenetics outcomes were evaluated |
| Hershey et al., Cephalalgia 2011 | Prospective observational study | Patients with chronic migraine and medication-overuse headache | Patients ≤ 18 years old |
| Corbelli et al., J Headache Pain 2012 | Prospective observational study | Patients with medication-overuse headache | No immunological/epigenetics outcomes were evaluated |
| Johnson et al., Headache 2015 | Double-blind, randomized, placebo-controlled pilot trial | Patients with medication-overuse headache | No immunological/epigenetics outcomes were evaluated |
| Bonnet et al., Nat Commun 2019 | Preclinical, mechanistic study | Mice with triptan-overuse headache | No immunological/epigenetics outcomes were evaluated |
| Wang et al., Eur J Neurol 2024 | Multicenter, real-world study | Patients with chronic migraine and medication-overuse headache | No immunological/epigenetics outcomes were evaluated |
| Wu et al., Ann Neurol 2024 | Cross-sectional study | Patients with chronic migraine and medication-overuse headache | No immunological/epigenetics outcomes were evaluated |

**Supplementary Table 4**. SYRCLE Risk of Bias (RoB) appraisal for included animal studies.

| **Domains** | Ryu et al. [12] | Zhang et al. [13] | Wang et al. [14] | Vuralli et al. [15] | Dağıdır et al. [16] | Urru et al. [24] |
| --- | --- | --- | --- | --- | --- | --- |
| Sequence generation | U | U | U | U | U | L |
| Baseline characteristics | U | U | U | U | U | L |
| Allocation concealment | U | U | U | U | U | U |
| Random housing | U | U | U | U | U | U |
| Blinding of caregivers/investigators | U | U | L | U | U | L |
| Random outcome assessment | U | U | U | U | U | U |
| Blinding of outcome assessor | L | L | L | L | L | L |
| Incomplete outcome data | U | U | U | L | L | L |
| Selective outcome reporting | U | U | U | U | L | L |
| Other sources of bias | U | U | U | U | L | L |

L: low risk of bias; U: unclear risk of bias.

**Supplementary Table 5**. Joanna Briggs Institute (JBI) critical appraisal tool for evaluating quasi-experimental studies.

| **Domains** | Carlsen et al. [20] | Mehta et al. [26] |
| --- | --- | --- |
| Cause-effect clearly identified | Yes | Yes |
| Participants similar across comparisons | NA/Unclear | Unclear |
| Co-interventions similar | Unclear | Unclear |
| Concurrent control group present | No | No |
| Multiple re/post measurements | Yes | Yes |
| Outcome measured in the same way | Yes | Yes |
| Outcome measured reliably | Yes | Yes |
| Follow-up complete/difference analyzed | Unclear | Unclear |
| Appropriate statistical analysis | Yes | Yes |
| **Overall risk of bias** | Moderate | Moderate |

NA: not applicable.

**Supplementary Table 6**. Joanna Briggs Institute (JBI) critical appraisal tool for evaluating cross-sectional studies.

| **Domains** | Ishii et al. [17] | Forcellini et al. [18] | Grazzi et al. [19] | Vuralli et al. [21] | Vuralli et al. [22] | Pisanu et al. [25] |
| --- | --- | --- | --- | --- | --- | --- |
| Inclusion criteria clearly defined | Yes | Yes | Unclear | Yes | Yes | Yes |
| Subjects and setting described in detail | Partial/Yes | Yes | Unclear | Yes | Yes | Yes |
| Exposure measured validly/reliably | Yes | Yes | Unclear | Yes | Yes | Yes |
| Standard criteria for condition used | Yes | Yes | Unclear | Yes | Yes | Yes |
| Confounders identified | No | Yes | No | Unclear | Unclear | Yes |
| Strategies for confounding stated | No | Partial/Yes | No | Unclear | Unclear | Yes |
| Outcome measured validly/reliably | Yes | Yes | Unclear | Yes | Yes | Unclear |
| Appropriate statistical analysis | Partial | Yes | Unclear | Unclear | Partial/Yes | Yes |
| **Overall risk of bias** | Moderate/High | Moderate | High | Moderate/High | Moderate/High | Moderate |

**Supplementary Table 7.** Joanna Briggs Institute (JBI) critical appraisal tool for evaluating randomized controlled trials.

| **Domains** | Karadaş et al. [23] |
| --- | --- |
| Allocation concealment | Unclear |
| Baseline comparability | Low risk |
| Blinding of participants/personnel | High risk |
| Blinding of outcome assessors | Low risk |
| Co-interventions/consistency of care | Low risk |
| Incomplete outcome data (attrition) | Unclear |
| Intention-to-treat analysis | Unclear |
| Outcome measurement | Low risk |
| Selective outcome reporting | Low risk |
| Other sources of bias | High risk |
| **Overall risk of bias** | High |
